# Earthworms newly from Mongolia (Oligochaeta, Lumbricidae, *Eisenia*)

**DOI:** 10.3897/zookeys.285.4502

**Published:** 2013-04-05

**Authors:** Robert J. Blakemore

**Affiliations:** 1 National Institute of Biological Resources (NIBR), Incheon, 404-708, Korea

**Keywords:** Far eastern Asian biodiversity, soil fauna, endemic vs.exotic invertebrates, Megadrilacea, climate extremes

## Abstract

Two new megadrile earthworms from the steppes, the first species wholly from Outer Mongolia, are ascribed to the partially parthenogenetic *Eisenia nordenskioldi* (Eisen, 1879) species-complex. Taxonomic justification of sympatric *Eisenia nordenskioldi mongol* and *Eisenia nordenskioldi onon* **ssp. n.** are supported by mtDNA COI barcodes. The unreliability of molecular differentiation based on voucher names compared to definitive types is again demonstrated, as pertains to the ultimate *Eisenia andrei* Bouché, 1972 synonym of the *Eisenia fetida* (Savigny, 1826) sibling species-complex composed of more than a dozen prior names. Similar species described from Northeast China [formerly Manchuria] and North Korea are briefly considered, albeit they are intermittently held in synonymy of cosmopolitan *Aporrectodea rosea* (Savigny, 1826) along with many other taxa including some exotic lumbricids initially found in India. Japanese and North American lumbricids are also mentioned. Distributions are discussed and an annotated checklist of all nine Siberian/sub-arctic *Eisenia nordenskioldi* ssp. is appended.

## Introduction

Holarctic family Lumbricidae continues to be refined, now providing approx. 670 valid taxa (plus ca. 55 uncertain species) from a total of 1,130 names in ca. 63 genera – or about 10% of all known megadrile earthworms – and contributing just 33 (or ~22%) of the 150 or so globally ubiquitous cosmopolitan species (Blakemore 2008a, 2010, 2012b). Natural distribution is from Vancouver Island in Canada, throughout Europe and Central Asia to Korea and Japan. Stephenson (1925) noted that no native species were known from Tibet or Mongolia, whereas Gates (1967 p. 172) concluded: “*In Manchuria, Kobayashi (1940) found that an annual rainfall of less than 400 mm (ca 16 inches) was unfavourable to earthworms. He likewise was mainly interested in native taxa. ‘In the region where the amount of annual rainfall is less than 400 mm, no endemic species can exist.’ (p. 308). Some at least, if not all, of the supposed endemics, when revised, will fall into synonymies of more or less widely spread anthropochores. Probably no single megadrile will prove to be autochthonous (evolved in, and not found elsewhere) in either Manchuria and Mongolia*”.

Prior to the current work, the only previous Mongolian record the author is aware of was for the giant *Eisenia magnifica* (Svetlov 1957: 183), formerly in genus *Allolobophora*, from the north-western Altai mountains bordering several countries thus its distribution is not restricted to Mongolia. A national report on sustainable development cites vermicompost production since 2005 by Ulziin Gol LLC a local company in Selenge Province, presumably using mundane compost-worm, *Eisenia fetida* (Savigny, 1826) that for the last 30 yrs, and currently, includes as its ultimate of 15 subsequent synonyms *Eisenia andrei andrei* Bouché, 1972 as determined by Easton (1983), Blakemore (2003, 2004 p. 97, 2006, 2008a, b, 2010, 2012a, b, 2013a, b), Csuzdi and Zicsi (2005 p. 143), Blakemore et al. (2010), Blakemore and Grygier (2011), and Csuzdi (2012).

## Materials and methods

Specimens, fixed in 75–80 % ethanol, lodged in National Institute of Biological Resources are available for transfer to a suitable Mongol national institute if regulations require. Description is in the author’s usual style (e.g. Blakemore 2010). Cytochrome c oxidase subunit 1 (COI barcode) sequences (Hebert et al. 2003) obtained using methods similar to those provided in Blakemore et al. (2010) are appended with analyses via megaBLAST (www.blast.ncbi.nlm.nih.gov/BLAST.cgi) and MEGA 5.1 (wwww.megasoftware.net) (Tamura et al. 2011). A checklist of boreal Palaearctic / Siberian *Eisenia nordenskioldi* species-complex, revised from those of Perel’ (1979, 1997), Easton (1983) and Blakemore (2004, 2008a, b), is presented in Appendix 2. Abbreviations are rhs- right hand-side, lhs – left hs; TP – tubercula pubertates; DP – dorsal pore; mid-D = mid-dorsal line.

## Systematic results

### Order Megadrilacea Benham, 1890

Family Lumbricidae Rafinesque-Schmaltz, 1815

Genus *Eisenia* Malm, 1877 [type-species *Enterion fetidum* Savigny, 1826]

#### Eisenia fetida

(Savigny, 1826) species-complex. s. Blakemore (2010)

http://species-id.net/wiki/Eisenia_fetida

Fig. 1

##### Note.

***Eisenia fetida***is the earliest representative of the genus, originally *Enterion fetidum* Savigny, 1826: 182 (type locality Paris; types in Muséum national d'histoire naturelle, Paris according to Stöp-Bowitz, 1969: 172); its 15 progressive synonyms, lastly including *Eisenia andrei andrei* Bouché, 1972: 381 (with types in Sully, France, OECO79-1388-4321), are fully presented in Blakemore (2008a, 2010, 2012, 2013a, b).

##### Material examined.

**Figure 1.**
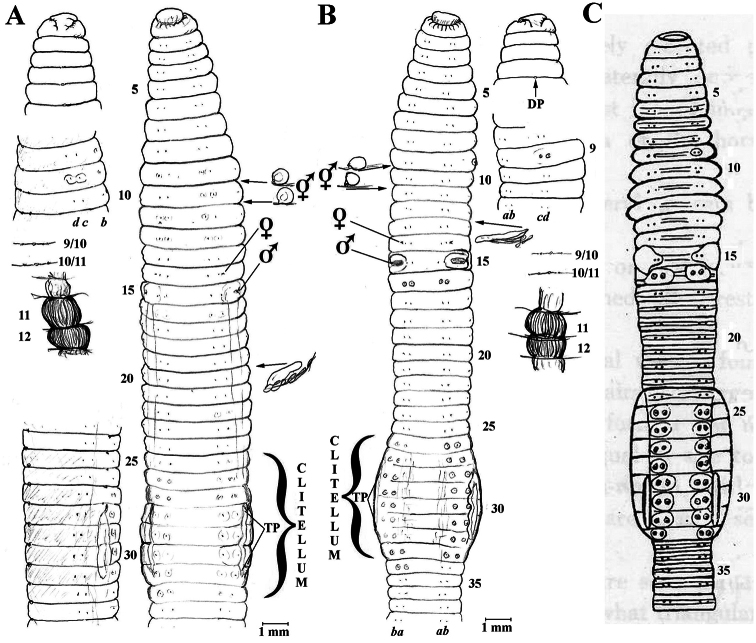
**A** *Eisenia fetida* specimen S1 from Jeju Isl., Korea; anterio-ventral and lateral views, dorsal prostomium; spermathecae and calciferous glands *in situ*, nephridium from 20lhs **B** *Eisenia fetida* S3 ditto with nephridium in 13lhs **C** Athecal *Allolobophora hataii* Kobayashi, 1940: fig. 5 (*incertae sedis*) for comparison.

Puce, semi-mature specimen S1 from Hamdeok Sewoobyong beach, Jeju Island, collected 15^th^ Feb., 2012 by RJB NIBRIV0000249915 (dissected and figured, Fig. 1, providing DNA sample WM18 – nil results, resampled as WO12 and as w11 to recheck); S2 mature, posterior amputee specimen with same collection data. S3-4 two uniformly pale Jeju specimens, collected 16^th^ Feb., 2012 by RJB (one posterior amputee dissected and figured, Fig. 1, providing DNA WO7 that was mixed in the genetics laboratory, resampled as w62 with data pending). S5 is a single deep-red, very weakly striped mature from Gangreung, Yongok stream, eastern S. Korea collected 4^th^ April, 2012 by RJB (IV0000249930 providing DNA sample WO18 – see Appendix 1). Three matures, pale with pink clitella, S6-8 from Incheon, Seo-gu, Gyeongseo-dong, 20^th^ April, 2000 (IV0000215368 mislabeled as “*Perionyx excavatus*”; note other *Perionyx excavatus* Perrier, 1872 proper confirmed in NIBR collection). Eight mature specimens, darkly striped with pale intersegments, otherwise compliant (IV0000261280 labeled “20110609//5/A” their jar also contains four *Amynthas* sp.). Other NIBR specimens labeled “*E. foetida*” e.g., IV0000213769/214062, were not inspected here.

##### Description of current specimens.

Body not especially flattened. Lengths 50-80 mm, segments 110–140. First dorsal pore small in 3/4, open from 4/5 onwards. Setae closely paired, *ab* slightly tumid in some or all of 9-12, 22, 23 and 25,26-32; distinctly paler around *cd* in just 9 or in some of 9-11,12. Dorsum to below *c* lines a reddish or pinky puce (sometimes much darker or much paler); ventrum pale with clitellum darker buff, saddle-shaped in 24,25,26,27-31,32,33. TP ½28,28-½31,31. Spermathecae nearly mid-dorsum in 9/10/11. Female pores small on 14 lateral to *b*. Male pores in slightly tumid pads on 15 lateral of *b* setae. Nephropores visible sporadically intersegmentally above *b* lines (alternatively in *d*?).

Internally, spermathecae spherical in 9 & 10. Testis small in 10 & 11, seminal vesicles in 9–12. Last hearts in 11. Calciferous glands annular in 11 & 12. Ovaries in 13. Nephridia sausage-shaped. Crop in 16 and gizzard large in 17-18, with intestine proper after 19; a low, wide typhlosole present from about 26.

**Remarks:** The Jeju specimens lack the supposedly characteristic broad striped appearance while specimen S5 is brick red (mtDNA barcodes show 99% agreement). It seems remarkable that S3-4 would agree as they lack pigment. Other specimens with much darker, almost black, segments and contrasting paler intersegment also comply superficially. The whole species-complex requires evaluation with consideration of ICZN compliance as noted in the Discussion.

### *Eisenia nordenskioldi*(Eisen, 1879) species-complex. *s*. Blakemore (2010)

(see Appendix 2)

#### Eisenia nordenskioldi mongol ssp. n.

http://species-id.net/wiki/Eisenia_nordenskioldi_mongol

Fig. 2 Tab. 1

##### Material examined.

**Figure 2.**
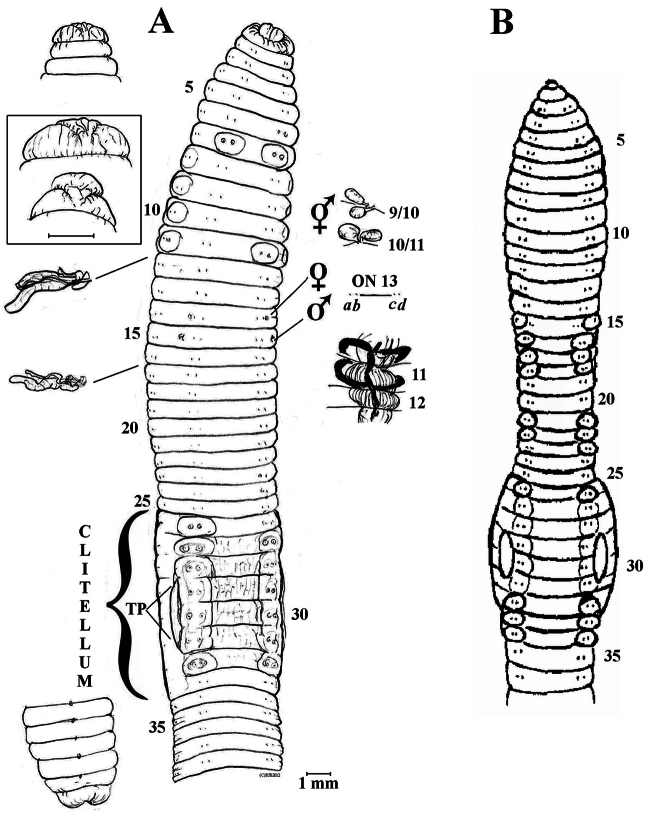
**A** *Eisenia nordenskioldi mongol* ssp. n. Holotype anterio-ventral view, dorsal prostomium [plus enlargements with that of P1 boxed], posterior, plus actual setal ratios on 13; spermathecae and calciferous glands *in situ*, nephridia in 12 & 17 **B** *Eisenia nordenskioldi polypapillata* after Perel’ (1969: text-fig) for fair use comparison.

Holotype (H), NIBR IV0000261274 (dissected and figured, Fig. 2, providing DNA - wo63); label details “*2012-7-22 Balji Riverside Coll. T-S Park*” (possibly near Onon-Balji Conservation Area) at Dadal (ca. 49°1'2.16"N, 110°37'18.49"E), Khentii Province NE of Ulaanbaatar, Mongolia. Paratype P1, IV0000261275 (dissected, providing DNA - wo64) plus six other specimens (P2-7, four mature, two sub-matures, IV0000261276) all labeled “*2012-7-21 Dadal*”.

##### Etymology.

Nominative singular noun in apposition, after natives of Mongolia.

##### Description.

Body substantial and only slightly trapezoid, posterior barely flattens. Pigment pinkish-grey dorsally in alcohol with ventrum and 9-11 paler laterally; clitellum buff. Lengths 80-110 mm (holotype H 60+50 = 110, paratype P1 80). Segments H 75+67 = 142, P1 131. Prostomium open epilobic (first thought tanylobic in H). Dorsal pores from 3/4 (minute), open from 4/5. Setae closely paired. Tumescences around setae *ab* on 7 & 11rhs plus 26lhs,27-32 (H); on 7 plus 27-33 (Ps); tumid and pale around lateral setae *cd* on 8-11 (H, P1). Clitellum saddle-shaped 26-33 (slightly encroaching onto 25 dorsally in some Ps). Tubercula pubertates faint, 29-31 lateral of setal *b* lines. Nephropores sporadically visible above *b* or *d* setal lines, e.g. above *d* in 9, 13, 14, 23-26, 34, 37, 38, 40, 41; or above *b* setal lines in some other segments in H. Spermathecal pores paired in 9/10/11 close to mid-D. Female pores in 14 lateral of *b*. Male pores small in 15 lateral of *b* just wider than female pores.

Internally, septa 8/9-10/11 slightly thickened. Spermathecae spherical on thin tapering stalks in 9 & 10. Testis and funnels non-iridescent (atrophied?) in 10 & 11. Seminal vesicles paired in 9-12 (smaller in 10). Ovaries compact in 13. Ovisacs vestigial anteriorly in 14. Hearts in 7-11. Nephridial bladders simple, sausage-shaped (in all segments inspected). Calciferous glands large and moniliform in 11 & 12. Crop in 15-16; muscular gizzard in 17-18 with septum 17/18 to midriff. Intestine proper from 19; slight typhlosole noticeably developing to inverted T-shaped from about 27,28. Gut contents mixed coarse organic material and some soil with mica flakes (i.e., a topsoil species). Apart from some loose gregarines, no parasites were observed in the coelom.

##### Remarks.

Lack of spermatozoal iridescence indicates parthenogenesis. *Eisenia nordenskioldi mongol* sub-sp. n. compares to the nominal subspecies and to *Eisenia nordenskioldi polypapillata* Perel’, 1969 differing from both due, at least, to its arrangement of setal tumescences. Kobayashi (1940 p. 282, 1941 p. 148) redescribed *Eisenia nordenskioldi* from Manchuria [=Northeast China] and northern Korea, while Zicsi (1972 p. 131) summarized *Eisenia nordenskioldi* from Pyongyang, North Korea. These taxa are compared in Table 1. Additionally, the DNA barcodes in Appendix 1 help define new and old taxa.

**Table 1.** Characters of *Eisenia nordenskioldi* sub-species after Kobayashi (1940), Perel’ (1969), Zicsi (1972) and pers. obs (cf. other sub-species in Appendix 2).

	***Eisenia nordenskioldi mongol* ssp. n.**	***Eisenia nordenskioldi polypapillata***	***Eisenia nordenskioldi nordenskioldi*** (*)
Length (mm)	80–110	55–80	25–45 (44–120*)
Segments	131–142	102–137	106–125 (101–176*)
Colour	Pink-grey	Pale	Dark puce to pale
Setae *aa:ab* ratio	Ca. 8–9:1	?	(7–8:1*)
Spermathecae	9/10/11 in mid-D	9/10/11 above *d*	9/10/11 mid-D
Papillae in *ab*	7(11), 25, 26–32	16–18, 21–23(24), 26 & 32–34	16, 22–34 (or 16, 22–34, 35 or just some*)
Paler tumid *cd*	8–11	Not noted	(10–12,13*)
Clitellum	½25,26–33	26,27–32	26,27–33
TP	29–31	½28,29-½31,31	29–31
Neph. bladders	Sausage-shaped	?	?
Typhlosole	Small T-shaped	?	?

*Features from Kobayashi’s (1940, 1941) descriptions compared to Zicsi’s.

#### Eisenia nordenskioldi onon ssp. n.

http://species-id.net/wiki/Eisenia_nordenskioldi_onon

Fig. 3

##### Material examined.

Holotype (H) NIBR IV0000261277 (mature, dissected, providing DNA sample - wo65) plus six sub-adults provisionally listed as paratypes (IV000061278) and a ‘tail’, all poorly-preserved in same batch from “*2012-7-20 Dadal*”. Unidentifiable were ca. 20 specimens (IV0000261279) some having clitella ca. 24-33 and TP ca. 28-30, also poorly-preserved from crowding in a single tube, labelled “*2012-7-21 Dadal*”. All specimens collected by NIBR’s Mr T.-S. Park.

##### Etymology.

Nominative noun in apposition after sample region where Dadal and the upper Onon River are supposed birthplace and likely final resting place of Temüjin (otherwise known as Genghis Khan).

##### Description.

Body medium sized, H 100 mm. Segments 170. Reddish pink anterior-dorsum to segment 15 otherwise unpigmented. Epilobous. Pale laterally around *cd* in 8-11 and slightly tumid *ab* on 11-12 and possibly somewhat on clitellum. First dorsal pore 4/5. Spermathecal pores in 9/10/11 mid-dorsally. Female and male pores slight, lateral of *b* setae on 14 and 15, respectively. Nephridia sporadically visible lateral of *b* lines near intersegments (at least on clitellum) otherwise near *d* lines? Clitellum, pale from 24 dorsally or laterally 25-33, i.e., 24,25-33 . TP longitudinally lenticular lateral of *b* 28-31. External features rather unclear due to poor preservation.

Internally similar to nominal subspecies. Seminal vesicles in 9-12. Testis iridescent, free in 10 & 11. Calciferous glands in 11 & 12, vascularized and extending slightly into adjacent segments. Nephridial bladders sausage-shaped. Gizzard 17-18 and thin inverted T-shaped typhlosole present. Soil with coarse organic debris in gut. No parasites were noted.

##### Remarks.

The current taxon differs from previously described subspecies (Tab. 1 and Appendix 2) on its clitellum, TP and tumescences; moreover it appears fertile. Fresher and better preserved material should confirm this analysis. In the meantime, although physically closest to *Eisenia nordenskioldi mongol*, it is clearly separated objectively on mtDNA data (Appendix 1). This compares to its sibling species-complex: European *Eisenia fetida* (Savigny, 1826) *vs*. *Eisenia andrei* Bouché, 1972 that is claimed to differ molecularly on enzyme gel electrophoresis, e.g. by Jaenike (1982) based on material from New York, but never yet on respective types of either taxon (see Appendix 1 and Discussion).

**Figure 3.**
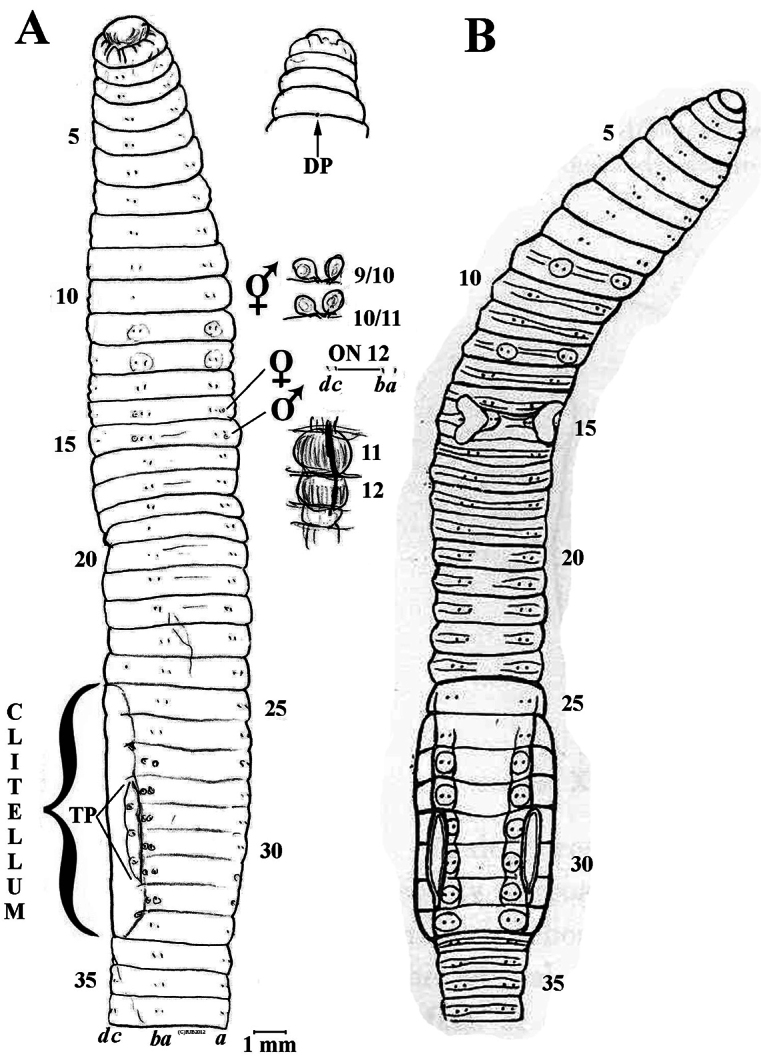
**A** *Eisenia nordenskioldi onon* ssp. n. Holotype sketched as for Figs **1–2** **B** *Allolobophora harbinensis* Kobayashi, 1940: fig. 6 (*incertae sedis*) for comparison.

## Discussion

Interest in natural and acquired species ranges intensifies with global climate concerns. Specific responses to extreme physico-chemical factors are also of interest. Lee (1985 p. 44) reports Ghilarov’s claim that *Eisenia nordenskioldi* revives after long periods of being frozen, with freeze tolerance down to -30°C recorded for *Eisenia nordenskioldi* (subspecies?) by Holmstrup and Petersen (1997) and Berman and Leirikh (1985). Berman et al. (2002) further report on adaptation to arid conditions. Its sibling species, *Eisenia fetida*, common at altitude in the Himalayas (Stephenson 1925), may be found in Spitzbergen or Siberia wandering on or under snow (some reports possibly misidentifications of *Eisenia nordenskioldi*?); and it is also found in deserts (e.g. of Arizona by Gates 1967) and Csuzdi and Pavlicek (2005a, b) recently report it from Mar Saba and Samaria, Israel and Jordan. *Eisenia fetida* was further located at hot springs on subarctic Iceland and a fumarole at subtropical Raoul Island, N.Z.; its experimental temperature range is given as -2° to +40°C (Lee 1985 tab. 2).

Regarding natural distributions of lumbricid earthworms and species identities, after synonymy of *Helodrilus (Bimastus) indicus* Michaelsen, 1907, Gates (1958 p. 6, 1972 p. 108) delineated the natural southern boundary of Lumbricidae in Asia north of the Hindu Kush and Karakorum ranges and from Baluchistan west to the Pacific. He thought endemicity of any lumbricid south of Tian Shan and Altai Mts (where giant *Eisenia magnifica* occurs) into Mongolia or Northeast China would be quite unexpected. Gates (1972 p. 108) said that his synonymy (in *Aporrectodea rosea*) was not accepted by all authors, indeed Easton (1983 p. 478) resurrected Michaelsen’s taxon as *Dendrobaena indica*, and whereas transfer was questioned by others (cf. genus *Healyella*), Dr Cs. Csuzdi (2003 pers. comm.) informed that “*I have seen the two type specimens. It seems a distinct species with unknown origin*”. Regardless of its generic status, *Dendrobaena indica* or *Helodrilus indicus* can no longer be thought to have been endemic to India, and neither is athecal Kashmiri *Allolobophora prashadi* (Stephenson, 1922) as noted below.

Although Perel’ (1969 p. 62) thought it likely that *Allolobophora harbinensis* Kobayashi, 1940 belonged in synonymy of *Eisenia nordenskioldi*, the characters Kobayashi (1940) provided showed similarity to his other three new species that were comparable to *Helodrilus (Allolobophora) prashadi* Stephenson, 1922: 440, another non-native from India and, after Gates (1958), usually placed in synonymy of *Aporrectodea rosea*. Kobayashi’s data are given in Table 2, albeit all five taxa are currently held in the extensive (four page!) synonym list of *Aporrectodea rosea* (Savigny, 1826) (e.g., by Gates 1974, Easton 1983, Blakemore 2008a, 2010, 2012; cf.Tab. 2). Quoting the generic definition by Michaelsen (1900 p. 471), Kobayashi (1940) presumably attributed his taxa to *Eisenia* as then defined only when the spermathecae were present and in or near the median-dorsal line, otherwise he put them into *Allolobophora* (including parthenogens?).

Possibly *Allolobophora harbinensis* is a sexual morph (and therefore an invalid synonym) of *Allolobophora hataii*. Alternatively, it may represent the amphimixic form of a separate taxon or, equally possible, they are subspecies of either of *Eisenia fetida* (most likely) or *onon nordenskioldi* but with spermathecal pores more lateral in *cd* lines. Nothing of substance separates Kobayashi’s *Allolobophora jeholensis* from his page prior *Allolobophora dairenensis* so it, at least, should be subsumed. Both have the flared clitella in 29-31 characteristic of *Aporrectodea rosea* and neither aresuperficially distinguishable from *Aporrectodea rosea* itself defined with clitellum in 25,26-31,32 and TP 29-½31,31 or thereabouts, plus several combinations of setal tumescences. Internally *Aporrectodea rosea* has spermathecae absent or in 9/10/11 dorsally; calciferous glands in 10; U-shaped nephridial bladders and it has a compound typhlosole – see Blakemore (2010, 2012). Thus possibly some or all of Kobayashi’s taxa, as well as athecal *Allolobophora prashadi*, may either be Northeast China candidates for *Aporrectodea rosea* or for parts of the *Eisenia fetida* and *Eisenia nordenskioldi* spp.-complexes. Interestingly, Kobayashi (1940 pp. 282-287) describes *Eisenia nordenskioldi* sub-species as well as both *Eisenia rosea* and *Eisenia fetida* from Northeast China! But, since he omits crucial morphological information (“?s” in Tab. 2), more work is therefore required for resolution of all Kobayashi’s taxa – extending to DNA analysis of primary types, if locatable and their DNA viable. More probably (topotypic) neotypes will be required – as per Blakemore et al. (2010) – to permit objective comparison with complete and correct identifications on GenBank notwithstanding. Such tasks far exceed the brief of the present study.

For *Eisenia nordenskioldi* spp-complex, Perel’ (1969) separated her *Eisenia nordenskioldi polypapillata* from the nominal type and a similarly unpigmented, *Eisenia nordenskioldi pallida* (Malevic, 1956) on the basis of its numerous papillae between the male pores and clitellum, and on the wider distance separating the spermathecal pores from the mid-dorsal line (Tab. 1). Dr Perel (pers. comm. Dec. 2012) now suspects both subspecies are variations of the same taxon, however this too would require reference to the earlier *pallida* and *acystis* types (if locatable).

As with *Eisenia fetida*, mere colour differentiation is probably inadequate. Kobayashi (1940), whose taxa were subsequently combined irrespective of their pigmentation, said typical *Eisenia nordenskioldi* somewhat resembled *Eisenia fetida* but were not quite so banded intersegmentally. In contrast, Zicsi (1972) noted his *Eisenia nordenskioldi* specimens reddish in life, when preserved were colourless. Thus wide intraspecific colour variations seem permissible in parts of *Eisenia nordenskioldi* too.

Some possibly similar species from the Siberian region are *Dendrodriloides grandis perelae* (Kvavadze, 1973) [syn. *Eisenia perelae polysegmentica* Kvavadze, 1979 (non Kvavadze, 1973)], *Eisenia sibirica* Perel & Graphodatsky, 1985, *Eisenia tracta* Perel, 1985, *Eisenia ventripapillata* Perel, 1985 and *Eisenia angusta* Perel, 1994. In the opinion of its author, *Eisenia ventripapillata* is certainly a separate species to *Eisenia nordenskioldi*; however, it is perhaps closer to *Eisenia acystis* (T. Perel pers. comm. via Anna Leirikh, 27^th^ Feb. 2013). The diagnostic comparison of *Eisenia ventripapillata* given was as an unpigmented worm with clitellum extending to 32/33 and TP occupying three segments at least from ½28 or 28, whereas in *Eisenia nordenskioldi* the TP is always from segment 29 (and clitellum to 33/34).

Another Siberian species claimed to be similar but separate from *Eisenia nordenskioldi* is *Eisenia atlavinyteae* Perel & Graphodatsky 1984: 611 (sometime spelt “*atlavynteae*”, “*atlaviniteae*” or “*atlavyntae*” and authored “Perel, Graf., 1985”). Vsevolodova-Perel and Bulatova (2008a, b) commented on polyploidy: “*Amphimictic autopolyploid races of two species of the Asian genus* Eisenia, E. nordenskioldi *and* E. atlaviniteae [sic, lapsus],* are widespread in Siberia, from its southern boundary to the arctic region, while polyploid Lumbricidae in the East-European plain, except for the Volga region, are represented mainly by parthenogenetic forms of other genera*.”

Polyploidy is often associated with parthenogenetic species complexes. Sexual forms of the *Eisenia nordenskioldi* species-complex are reported to have even ploidy levels (orthoploids with 2× being equal to 36 rather than 24 according to Bulatova et al., 1987) ranging from 2n-8n, while the only previously recorded parthenogen is a deep-burrowing and athecal septaploid (7n), *Eisenia nordenskioldi acystis* (Michaelsen, 1903) (with 10× = 110-l15) found in the Talasskii Alatau mountains (Perel and Grafodatsky 1983) that Viktorov (1997) called a “*race*” (and, if so, an invalid taxon). Other taxa like *Eisenia nordenskioldi pallida* may be di- or tetraploid, and ecological differences of polyploid vs. diploid morphs shows wide distribution and variation: the more wide, the higher the ploidy level (Perel’ 1987, Grafodatsky et al. 1982, Perel and Bulatova 2008a, b).

Regarding distribution of the species-complex, Kobayashi (1940, 1941) found *Eisenia nordenskioldi nordenskioldi* to be prevalent in the DongBei Region of China and North Korea; differentiating his more darkly pigmented *Eisenia nordenskioldi manshurica* subspecies (Appendix 2 cf.Tab. 1) that he also thought similar to Caucasian *Eisenia nordenskioldi lagodechiensis* (Michaelsen, 1910) but lacking its glandular male pores, as do all the other sub-species. Dr Perel’s *Eisenia nordenskioldi polypapillata* is from the Dzungarian Alatau mountain range at Almaty Province of south-eastern Kazakhstan. The current species are from much further east in Mongolian river tributaries flowing from the Khentii Mountains to the steppes.

Perel’ (1987) states: “..Eisenia nordenskioldi *in southern Kazakhstan, Siberia and the Far East is represented by the poorly pigmented form* pallida.* The typical form is significantly more widespread, in Siberia reaching the regions of the far north and also occurring in the eastern and south-eastern parts of the European USSR”* and Perel’ (1997 p. 22) gives the location of dubious *Eisenia nordenskioldi pallida* morph or subspecies in “..Китая и на севере Корея.” (= China and in northern Korea). These citations by Perel (1979 pp. 75, 267, 1997 pp. 69, 70) may be mistaken if priority yields to *Eisenia acystis* (Michaelsen, 1903), thus leaving *Eisenia nordenskioldi pallida* Malevics, 1956 as *species inquirendum*. Historical reports of the nominal taxon from the Azores and Hawaii are probable misidentifications with *Eisenia fetida* (as noted by Michaelsen, 1900 p. 476); while Garman (1888 p. 73) said that *Eisenia nordenskioldi* was: “*Obtained by Eisen in Siberia; credited to North America by Vejdovsky*”, i.e., its USA credit was mistaken too.

Confusion between these sibling species may have been common. Both Michaelsen (1903, 1910) and Gates (1972 p. 103) recognized variability of (parthenogenetic and/or polyploidal) morphs and close relationships of Siberian *Eisenia nordenskioldi* (Eisen, 1879) with European *Eisenia fetida* (Savigny, 1826), Gates saying they were “*indistinguishable specifically from each other by any of the characters of the classical system*” and differing substantially only in the number of atyphlosolate posterior segments. The whole *Eisenia fetida* species-complex yet requires evaluation with consideration of ICZN compliance. For example, as noted above, Jaenike (1982) avoided types and overlooked the synonyms with priority over *Eisenia andrei*, the first being *Eisenia semifasciatus* (Burmeister, 1835) which has not yet been tested and neither have any of Kobayashi’s species as noted herein. Moreover, at least Stop-Bowitz (1969, tab. V) maintains Scandinavian *Eisenia fasciata* Backlund, 1948 which is often included in *Eisenia fetida* synonymy by most authors along with ca. 14 other names, but more often than not (especially in chemical/molecular studies by non-taxonomists) these available synonyms are completely overlooked (see also the discussion in Blakemore et al. 2010).

This notion, that components of the *Eisenia fetida* and *Eisenia nordenskioldi* spp. complexes are indistinct, is gradually being falsified by refined genetic information complementing the morphology of taxa under rules of ICZN (1999) that disallows nomenclatural availability to varietal forms, morphs or races. However, further considerations are, firstly, that genetics only reveals a part of the information on a taxon while a morphological character is often controlled and manifest from interplay of several genes throughout the organism’s ontogeny and phylogeny (with ontogeny defined as the history of structural change in any biotic entity whether a cell, an organism, or a population of organisms, i.e., a species). Secondly, regardless of data being based on DNA or morphology, or on both of these, it is only the condition pertaining to the ICZN (1999) defined type-specimen that defines the scientifically-named species. Hence a chronic confusion of all *Eisenia fetida/andrei* results – see Blakemore (2006, 2010, 2012b, 2013a, b) and Blakemore et al. (2010), the latter while also providing a model from the first COI barcode of an earthworm’s neotype, comments on the shortcomings of all previous molecular studies. Just as Blakemore (2011) observed regarding a New Zealand paper: “*as with several previous molecular phylogenetic works, the only errors in their otherwise informative study are the names*”.

The genus is a contrivance itself defined by its type-species’ type; ditto a family.

**Figure 4.**
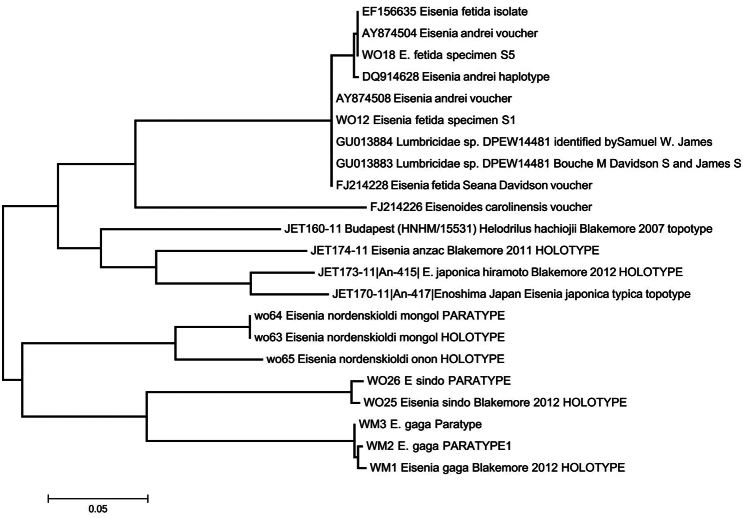
MEGA 5.1 default NJ-ML phylotree of COI barcodes (with sequences aligned using the Clustal X option defaults and S1 (WO12), S5 (WO18) and *Eisenia gaga* complements reversed) showing unreliability of GenBank (blast.ncbi.nlm.nih.gov/genbank) and/or Bold Systems (boldsystems.org) voucher names compared to eloquent power of barcoding definitive ICZN 1° types. Cf. data in Appendix 1.

Despite morphological limitations (Tabs 1–2), objective DNA data (Appendix 1) and regulated ICZN taxonomy (Appendix 2) complement comfortably herein (Fig. 4).

**Table 2.** Similar *Allolobophora* species (or rather parthenogenetic morphs?) as described by Kobayashi (1940) with characters he used for separatation bolded.

	***Allolobophora hataii* Kob., 1940: 288**	***Allolobophora harbinensis* Kob., 1940: 290**	*Allolobophora dairenensis* Kob., 1940: 291 (*)
Length (mm)	78–97	76–96	80–111 (41–53*)
Segments	134–142	134–144	137–139 (132–140*)
Prostomium	Pro-epilobous	Pro-epilobous	Pro-epilobous
Colour	Grey	Grey	**Pinkish** (Pale*)
Setae *aa:ab* ratio	96:7 (post-clit.)	93:7.5	83:8 (40:3.8*)
1^st^ dorsal pore	4/5/6	4/5	4/5
Spermathecae	**Absent**	9/10/11 in *cd*	**Absent**
Papillae in *ab*	(9)15, 16, 25–32	9, 12, 27–32	9, 15, 16, 23–33
Paler tumid *cd*	10–12	9, 10, 12	10–12 (9–12*)
Clitellum	24–32,33	25,26–32,33	**23–33** (23–32,33*)
TP	29–31	29–31	29–31
Male pores	Prominent	**Horseshoe-shape**	Prominent
Neph. Bladders	?	?	?
Ca Glands	?	?	?
Typhlosole	?	?	?

*For *Allolobophora jeholensis* Kob., 1940: 293 that differs inconsequentially from *Allolobophora dairenensis*.

## Supplementary Material

XML Treatment for Eisenia fetida

XML Treatment for Eisenia nordenskioldi mongol

XML Treatment for Eisenia nordenskioldi onon

## Appendix 1

**DNA COI and BLAST analysis**

Confidently proven barcode results will eventually be uploaded to GenBank (www.blast.ncbi.nlm.nih.gov/genbank) and/or Bold Systems (www.boldsystems.org).

[>WO7 *Eisenia fetida* specimen S3 from Jeju Island, Korea. Sample mixed/contaminated – megaBLAST result 99–100% *Aporrectodea trapezoides*, or an unidentified French “*Lumbricidae sp. DPEW31891*” No. GU013952; this result identical with samples WO13 & WO14 I identified as *Bimastos parvus* from Jeju and *Aporrectodea trapezoides* species-complex specimens from NZ, respectively; these obviously require redoing as noted in Blakemore (2013a)].

>WO12 *Eisenia fetida* specimen S1 from Jeju Island, Korea collected by RJB.

ATAAATGTTGGTAGAGAATAGGGTCGCCACCTCCAGCAGGGTCAAAGAATGAGGTATTTAGGTTTCGATCTGTCAATAGTATAGTGATAGCTCCCGCAAGTACTGGAAGAGATAAAAGTAGTAACACCACGGTAATAACTACAGCTCATACAAATAGGGGGATTCGTTCTAGTCGAAGCCCACTTCATCGTATGTTAATAACTGTAGTAATGAAGTTAATTGCCCCTAAAATTGAGGAGGCACCTGCTAAATGGAGGGAAAAAATAGCCAGGTCCACTGAGGGCCCCGCGTGCGCTAAGTTACTGGATAGGGGTGGGTAAACTGTCCACCCTGTTCCAGCACCCTTTTCCACTGCAGCAGAGGATACTAGGAGAATTAGGGAAGGGGGCAGAAGTCAAAATCTTATGTTGTTGAGACGTGGAAAGGCTATGTCTGGAGCTCCTAGTATAAGAGGTAGAAGTCAGTTTCCAAATCCACCAATAAATACAGGTATTACCAGAAAGAAAATTATTACAAATGCATGGGCTGTAACAATTGTATTATATAGTTGGTCCCTTCCTAGGAAGGCACCTGGTTGCCTTAGCTCGATTCGAATGAGAAGGCTTATACCAGCACCAACCATACCTGCTCAGACCCCGAGAATGAAATAGAG

megaBLAST – 100% for “*DNA barcodes for soil animal taxonomy: transcending the final frontier*” by Bouche & James as unidentified “*Lumbricidae sp*.”!, or 100% *Eisenia fetida*, or 100% *Eisenia andrei* by Perez-Losada, *et al*. 2005; GenBank Nos. , GU013883.1, FJ214228.1, AY874508.1, none being based on valid types; or 99% for “*Eisenia andrei* haplotype” DQ914628.1 this again not an ICZN valid type designation.

Recheck analysis as w11 yielded exactly same - nBLAST Id = 617/617 (100%). QED.

>WO18 *Eisenia fetida* specimen S5, 4^th^ April, 2012 mainland Korea collected by RJB.

TCAGAATAAATGTTGGTAGAGAATAGGATCGCCACCTCCAGCAGGGTCAAAGAATGAGGTATTCAGGTTTCGATCTGTCAATAGTATAGTGATAGCTCCCGCAAGTACTGGAAGAGATAAAAGTAGTAACACCACGGTAATAACTACAGCTCATACAAATAGGGGGATTCGTTCTAGTCGAAGCCCACTTCACCGTATGTTAATAACTGTAGTAATGAAGTTAATTGCCCCTAAAATTGAGGAGGCACCTGCTAAATGGAGGGAAAAAATAGCCAGGTCCACTGAGGGCCCCGCGTGCGCTAAGTTACTGGATAGGGGTGGGTAAACTGTCCACCCTGTTCCAGCACCCTTCTCCACTGCAGCAGAGGATACTAGGAGAATTAGGGAAGGGGGCAGAAGTCAAAATCTTATGTTGTTGAGACGTGGAAAGGCTATGTCTGGAGCTCCCAGTATAAGAGGTAGAAGTCAGTTTCCAAATCCACCAATAAATACAGGCATAACCAGAAAGAAAATTATTACAAATGCATGGGCTGTAACAATTGTATTGTATAGTTGGTCCCTTCCTAGGAAGGCACCTGGTTGCCTTAGCTCGATTCGAATGAGAAGGCTTATACCAGCACCAACCATACCTGCTCAGACCCCGAGAATGAAATAGAGA

megablastBLAST –100% *Eisenia andrei* by Perez-Losada et al. 2005, or 100% *Eisenia fetida* from USA, or 99% “*DNA barcodes for soil animal taxonomy: transcending the final frontier*” by Bouche & James an unidentified French “*Lumbricidae sp*.” with GenBank Nos. AY874504.1, EF156635.1, GU013884.1 – none valid ICZN types.

nBLAST WO12 vs. WO18 Id = 644/652 (99%), i.e., tolerably the same taxon.

>wo63 *Eisenia nordenskioldi mongol* H.

CATAGTAGGTGCAGGAATAAGACTTCTCATCCGAATTGAATTAAGTCAGCCGGGTGCCTTCCTAGGTAGAGATCAACTATACAACACAATTGTCACAGCTCACGCCTTTGTGATAATCTTCTTCTTAGTTATACCTGTATTTATTGGGGGATTTGGAAACTGACTCCTTCCTCTAATACTAGGTGCCCCTGACATAGCCTTTCCTCGTCTTAATAACATAAGCTTCTGACTTCTACCCCCCTCCCTAATCCTACTAGTATCCTCTGCCGCAGTAGAAAAAGGAGCTGGCACAGGATGAACTGTATACCCTCCCTTATCTAGGAATATTGCCCATGCTGGCCCTTCAGTAGATTTAGCAATTTTTTCCCTACATTTAGCTGGAGCTTCATCAATTCTTGGTGCTATTAACTTTATCACCACAGTAATTAATATGCGGTGAACAGGTATACGTCTCGAACGAATCCCTCTATTTGTCTGAGCTGTAATTATCACAGTGGTCTTACTTCTTCTTTCTCTTCCGGTTCTTGCAAGAGCCATTACCATACTTCTGACAGACCGAAACCTCAATACTTCATTTTTTGATCCTGCTGGAGGGGGGGACCCTATCCTTTACCAGCA

megaBLAST max id for random lumbricids =<83%, *i.e*., new relative to GenBank. nBLAST wo63 *vs*. WO12 *Eisenia fetida* Id = 497/617 (81%), i.e., clearly different spp.

>wo64 *Eisenia nordenskioldi mongol* P.

GTACTCTTTACTTTATTCTAGGCGTCTGGGCCGGCATAGTAGGTGCAGGAATAAGACTTCTCATCCGAATTGAATTAAGTCAGCCGGGTGCCTTCCTAGGTAGAGATCAACTATACAACACAATTGTCACAGCTCACGCCTTTGTGATAATCTTCTTCTTAGTTATACCTGTATTTATTGGGGGATTTGGAAACTGACTCCTTCCTCTAATACTAGGTGCCCCTGACATAGCCTTTCCTCGTCTTAATAACATAAGCTTCTGACTTCTACCCCCCTCCCTAATCCTACTAGTATCCTCTGCCGCAGTAGAAAAAGGAGCTGGCACAGGATGAACTGTATACCCTCCCTTATCTAGGAATATTGCCCATGCTGGCCCTTCAGTAGATTTAGCAATTTTTTCCCTACATTTAGCTGGAGCTTCATCAATTCTTGGTGCTATTAACTTTATCACCACAGTAATTAATATGCGGTGAACAGGTATACGTCTCGAACGAATCCCTCTATTTGTCTGAGCTGTAATTATCACAGTGGTCTTACTTCTTCTTTCTCTTCCGGTTCTTGCAAGAGCCATTACCATACTTCTGACAGACCGAAACCTCAATACTTCATTTTTTGATCCTGCTGGAGGGGGGGACCCTATCCTTTACCAGCACT

nBLAST “H” *vs*. “P” Id = 618/618 (100%), i.e., ostensibly the same taxon. QED.

>wo65 *Eisenia nordenskioldi onon* H.

GTTTGGGCCGGCATAGTGGGTGCCGGAATAAGACTTCTTATCCGAATTGAGTTAAGTCAGCCGGGAGCCTTTCTAGGCAGAGATCAACTATATAATACAATTGTTACAGCTCACGCCTTTGTAATAATCTTCTTCTTAGTTATACCTGTATTTATTGGAGGATTTGGAAACTGACTTTTACCTCTAATACTAGGTGCCCCTGATATAGCCTTTCCTCGTCTAAATAACATAAGCTTTTGACTTCTACCCCCCTCCCTAATCCTCCTAGTTTCCTCTGCCGCAGTTGAGAAAGGAGCTGGCACAGGATGAACTGTATACCCCCCCCTATCTAGAAATATTGCCCATGCTGGCCCTTCCGTAGATTTAGCAATTTTTTCGCTACATTTAGCCGGAGCTTCATCAATTCTTGGAGCTATTAACTTCATCACCACAGTAATTAATATACGATGAGCAGGTATACGTCTTGAACGAATCCCTTTATTTGTCTGAGCTGTGATTATTACAGTAGTCTTACTTCTACTTTCTCTCCCGGTGCTGGCAGGAGCTATTACCATACTTCTAACAGACCGAAACCTTAATACTTCATTTTTTGATCCTGCTGGTGGGGGGGACCCTATCCTATATCAACACCTTTTT

megaBLAST max. alignment for random lumbricids =<84%, i.e., again nothing similar yet on GenBank but future comparisons with this definitive type now possible.

nBLAST wo65 *vs*. wo63 Id = 562/609 (92%), i.e., ostensibly different (sub-)species.

All above sequences and megaBLAST results are compared in a MEGA 5.1 default phylotree (Fig. 4) against Japanese *Eisenia japonica* (Michaelsen, 1892) plus some of its sibling species, and against FJ214226 *Eisenoides carolinensis* (Michaelsen, 1910) from USA as the author discusses in Blakemore and Park (2012). Specifically, all *Eisenia andrei* Bouché, 1972 records may be dismissed or falsified in favour of any of the 14–15 prior synonyms of *Eisenia fetida* (Savigny, 1826) that are thoroughly detailed in Blakemore (2004, 2008, 2010, 2013) and are cogently discussed in Blakemore et al. (2010). As Blakemore (2011) observed in relation to a New Zealand study: “as with several previous molecular phylogenetic works, the only errors in their otherwise informative study are the names.”

## Appendix 2

**Annotated checklist of *Eisenia nordenskioldi* species-complex (chronological)**

1. *Eisenia nordenskioldi nordenskioldi* (Eisen, 1879: 6). [Emend. corr. from *nordenskiöldi* e.g. by Blakemore (2008a p. 39); Perel (1979 p. 218) originally included *Allolobophora acystis* in synonymy; but she later stated (Perel 1997 pp. 69–71) that the previous “*Eisenia acystis* Michaelsen, 1903” synonym was erroneous. Miscited as “*nordenskioeldi*” by Blakemore (2004 p. 98) after Easton (1983 p. 480) from original spelling of “*nordenskiöldi*” and as per Michaelsen (1900 p. 476), since it is named after the famous explorer – Baron Nils Adolf Eric Nordenskiöld (1832–1901) – the ‘ö’ is Finnish and not a German umlaut so was corrected under ICZN (1999 Art: 32.5.2.1) with just the diacritic removed. Alternate original spelling “*nordenskiöldii*” Eisen (1879: 46). Name sometimes misspelt “*nordenskioeldii*”, “*nordenskjoldii*”, “*nordenscioldi*”, “*nordenskiöldii*”, or “*nordenskjöldi*” e.g. by Reynolds and Cook (1976 p. 145); misquoted as “Eisen, 1873” in Perel’ (1997 p. 69)]. Furthermore, Reynolds and Cook (1976 p. 85) cite a taxon: “*Eisenia nordenskjöldi* var. *caneasia* Mich., 1907: 82” (sic), probably a mistake for *Eisenia nordenskioldi caucasica* that they omit].

2. *Eisenia nordenskioldi caucasica* Michaelsen, 1903: 38 ***species inquirendum*** [non *Dendrobaena caucasica* Kulagin, 1889 (= *Dendrobaena veneta*)]. [Often misdated “1902” as with *acystis*. From Transcaucases, to 2,500 m altitude (Michaelsen, 1903 p. 39) found also in association with *Eisenia fetida*; its clitellum is 24,25-33 and TP 27,28-31 or 28-½32 . Although Michaelsen has types in St. Petersberg they are listed in Hamburg (6959) by Reynolds and Cook (1976 p. 85) for their “*Ei. nordenskjöldi caneasia* Mich., 1907: 82” (sic, lapsus pro *caucasica*) in a 1907 Georgian publication that seems not to exist. Easton (1983 p. 480) at least placed *caucasica* in synonymy of *Eisenia fetida*, although this placement requires re-evaluation with the benefit of recent taxonomic advances (e.g., DNA and IBM)].

3. *Eisenia nordenskioldi acystis* (Michaelsen, 1903: 43) ***species inquirendum***. [Originally *Helodrilus acystis*; ?non “*Haplotaxis acystis* Michaelsen, 1903: 43” (lapsus?) – this taxon, often repeated on species databases, has the same publication data and is likely a mistake]. [A parthenogenetic sub-species of some part of the species-complex lacking spermathecae (and spermatophores?); it was unpigmented, described with clitellum in 26-33 and TP in 29-½31; seminal vesicles in 9-12; *cf*. *Eisenia nordenskioldi pallida*. From Turkestan with types in St. Petersburg Museum. Dr T. Perel (pers. comm. via A. Leirikh 27^th^ Feb. 2012) now considers it a separate taxon from *Eisenia nordenskioldi*. Note: Its publication date is confirmed as 1903 since the journal states “*Augegeben am 27. Mai 1903*” rather than “1902” as scheduled].

4. *Eisenia nordenskioldi lagodechiensis* (Michaelsen, 1910: 18) ***species incertae sedis***. [Originally in *Helodrilus*; returned to separate species level by Kvavadze (1993) because its developed male pores are apparently distinctive (cf. Kobayashi’s, 1940 taxa). From Georgia, the types probably in St Petersburg].

5. *Eisenia nordenskioldi manshurica* Kobayashi, 1940: 284 ***species inquirendum*.** [Sometimes included as a synonym of nominal sub-species, its characters probably justify its separation: Dark pigment. Length 111-144 mm, segments 154-175), clitellum in 26,27-34 and TP 29-32. Type-locality is likely Anshan, where two clitellate specimens were found (August, 1938) along with two aclitellate specimens; other semi-clitellates from Chinhsien and Mutanchiang but all syntypes are now lost following Pacific and Korean wars].

6. *Eisenia nordenskioldi pallida* (Malevic, 1956: 439) ***species inquirendum*** (non *Allolobophora pallida* Bretscher, 1900: 41). [Cited in Perel’ (1969 p. 62, 1979 pp. 75, 267) and Vsevolodova-Perel (1997 pp. 69-71) as *Eisenia nordenskiöldi pallida* Malevic, 1956 latterly with restoration of synonym *Eisenia acystis* (Michaelsen, 1903) - this possibly a mistake as the priority would yield to *Eisenia acystis* with *acystis* itself possibly remaining a synonym of the nominal subspecies. Both taxa overlooked by Reynolds and Cook (1976) and by Easton (1983). The original reference (listed as “Ucs. Zapisk. M. Gor. Ped. Inst. 61: 439-448” on www.earthworm.uw.hu) was inaccessible at time of current submission].

7. *Eisenia nordenskioldi polypapillata* Perel, 1969: 61 ***species inquirendum***. [Its papillae and position of spermathecae, as originally described, are perhaps definitive (Tab. 1). Collected in May, 1967 from “*Dsungar-Ala-Tau, Bezirk Sarkand, Försterei Topolevskoje*” (a forestry station in Sarkand district of the Dzungarian Alatau mountain range at Almaty Province of south-eastern Kazakhstan); found in Apple and Fir forest at 1,200–1,500 m altitude. Holotype and Paratype from ten poorly-preserved specimens in Zoological Museum of Moscow University, No. W-10. Dr Perel now suspects it is synonymous (pers. comm. via Anna Leirikh Dec. 2012 ) “*She also now suppose that* E. n. polypapillata *in fact is* E. n. pallida,* and some authors considers* E. n. pallida *as separate species*”; however the relationship of both to prior *Eisenia nordenskioldi acystis* remains unclear as noted above].

8. *Eisenia nordenskioldi mongol* Blakemore, 2013 ssp. n. with its DNA data provided.

9. *Eisenia nordenskioldi onon* Blakemore, 2013 ssp. n. with its DNA data provided.
